# Mixture–Process Design and Multiresponse Optimization of Starch–Gelatin Films Containing Nopal-*Opuntia* spp. Mucilage: Mechanical Properties and Apparent Mass Loss

**DOI:** 10.3390/polym18202528

**Published:** 2026-10-15

**Authors:** Juan Paredes, Germán Flores, Cristian Pérez-Salinas, Gabriela Salinas

**Affiliations:** 1 GI3M—Grupo de Investigación e Innovación en Ingeniería Mecánica, Universidad Técnica de Ambato, Ambato 180104, Ecuador; jgparedes@uta.edu.ec (J.P.); cf.perez@uta.edu.ec (C.P.-S.); 2 Facultad de Ingeniería Civil y Mecánica, Universidad Técnica de Ambato, Ambato 180104, Ecuador; jgparedes@uta.edu.ec (J.P.); germanrf486@gmail.com (G.F.); cf.perez@uta.edu.ec (C.P.-S.); 3 Facultad de Ciencias de la Salud y Bienestar Humano, Universidad Tecnológica Indoamérica, Ambato 180212, Ecuador; veronicasalinas@uti.edu.ec

**Keywords:** biopolymer films, starch–gelatin blends, nopal-*Opuntia* mucilage, mechanical properties, apparent mass loss, multiresponse optimization, desirability function

## Abstract

The performance of biopolymer films depends on both composition and formation conditions. This study evaluated films containing constant amounts of Nopal-*Opuntia* spp. mucilage, glycerol, and vinegar using a 16-run mixture–process design with four starch–gelatin ratios and post-drying thermal-treatment settings of 40 or 60 °C for 5 or 10 min. Maximum tensile stress, a provisional modulus, elongation, and apparent mass loss in soil and outdoors were analyzed over 48 days. Model adequacy was assessed using fit statistics, leave-one-formulation-out predictions, and influence diagnostics. The primary multiresponse solution assigned lower relative importance to the two gravimetric responses than to the three mechanical responses and corresponded to starch/gelatin 0.25/0.75 at 40 °C for 5 min (D = 0.644), coinciding with observed run F04. Equal-response and gravimetric-loss-emphasis solutions were retained as sensitivity analyses; their intermediate times are unconfirmed model interpolations. F04 showed the highest observed stress (1.614 MPa) and provisional modulus (24.358 MPa), F16 the greatest elongation (99.269%), and F09 the greatest observed apparent mass loss (93.0% in soil and 95.0% outdoors). One film was manufactured per run, the starting mass used for gravimetric calculations was nominal, and no confirmatory runs were conducted. Accordingly, the estimates remain formulation-specific and exploratory; mass loss does not demonstrate biodegradation.

## 1. Introduction

Biopolymer-based films provide a versatile platform for developing short-service-life materials with tunable compositions and performance. However, partially replacing conventional matrices involves more than selecting a renewable feedstock. Final performance emerges from interactions among composition, plasticizer, film-forming solution preparation, and post-drying thermal treatment. In starch-based films, these relationships produce potentially competing responses, including strength, stiffness, deformability, solubility, and water stability [1,2,3,4]. Formulation and processing should therefore be investigated jointly rather than treating temperature and time as merely operational parameters.

Starch is widely available and has film-forming capacity, but films prepared solely from this biopolymer are often brittle and moisture-sensitive. Gelatin can improve network continuity and modify tensile stress, elastic modulus, and elongation; nevertheless, the direction and magnitude of its effect depend on the starch–gelatin ratio, starch source and structure, plasticizer, moisture, and conditioning [3,4,5,6]. Starch–gelatin systems have exhibited both strength gains associated with amylose content and pronounced increases in deformability at higher gelatin fractions, together with compositional regions of limited compatibility [4,5]. These findings are not contradictory when the differences in matrices, proportions, and experimental conditions are recognized.

Glycerol increases chain mobility and promotes flexibility, but it can reduce strength and stiffness when it weakens intermolecular interactions. In *Opuntia ficus-indica* mucilage films, polyol identity significantly affected mechanical and barrier properties [7]. Early systematic studies of *Opuntia* mucilage films further showed that pH, calcium, and extraction procedure alter film structure and mechanical behavior [8,9]. In potato-starch matrices containing nopal mucilage, the combination of composition and drying temperature also affected the film’s physicochemical properties [10]. Consequently, mucilage should not be treated as an additive with a constant effect; its contribution depends on the matrix and processing history.

The literature confirms that cactus mucilage can be combined with proteins and other polysaccharides through markedly different strategies. Films have been developed from *Cylindropuntia fulgida* mucilage and gelatin [11], *O. ficus-indica* mucilage with gelatin and glycerol or sorbitol [12], cactus mucilage with agar [13], and *Opuntia* mucilage with poly(vinyl alcohol) [14]. Calcium-treated single-biopolymer films of mucilage, pectin, and alginate have also been compared [15]. Collectively, these studies show that strength, modulus, and elongation depend on the complete network architecture and that results obtained for a particular species or blend cannot be transferred directly to another formulation.

Recent studies have extended these approaches through mineral fillers, nanofibers, and mucilage treatments. Teshager et al. incorporated acid-leached kaolin into *O. ficus-indica* mucilage films [16], whereas Shinga et al. optimized glycerol and cellulose-nanofiber concentrations by response surface methodology [17]. Monteiro et al. combined fermented *O. cochenillifera* mucilage with corn starch and evaluated the mechanical and functional properties [18]. Other studies incorporated mucilage into styrene–butadiene latex [19] or PVOH [20] matrices and related mechanical characterization to environmental tests. Gelatin films containing *Opuntia* products have also been studied [21], and starch–gelatin films reinforced with Laponite^®^ have recently been assessed for thermomechanical behavior and aerobic biodegradability [22]. These reports confirm the current relevance of the topic but also show that responses depend on composition, reinforcement, processing, and the method used to evaluate environmental behavior. Because no verifiable species-level taxonomic identification was available for the present study, the plant material is conservatively designated *Opuntia* spp.

Designs of experiments can model these dependencies, but the use of this approach alone does not constitute novelty. Constrained mixture designs have been applied to starch and other biopolymer films [23,24], Bayesian optimization to thermoplastic starch films [25], and combinations of ANOVA, mixture design, and multivariate analysis to starch–glycerol–carboxymethylcellulose films [26]. Magnaghi et al. specifically used a combined mixture–process design and multicriteria optimization [1], whereas Araujo-Farro et al. jointly studied glycerol concentration, pH, and drying time–temperature conditions [2]. Starch–gelatin matrices have likewise been optimized using response surface methods, simplex-lattice designs, and multiresponse criteria [3,4]. Within this framework, the desirability function converts each predicted response into an individual desirability and combines them into a composite indicator whose value depends on analyst-defined limits, targets, weights, and importances [27]. The scientific value of optimization therefore lies in making property trade-offs explicit and distinguishing the best observed runs from continuous solutions predicted by the models.

Environmental assessment requires additional conceptual caution. Mechanical properties of Opuntia-containing films have been paired with soil burial, water exposure, and other gravimetric tests [19,20,21,22]. In a different system, Akhir et al. compared PBAT–thermoplastic starch films under soil burial and natural weathering and showed that the exposure environment affects material mechanisms and evolution [28]. Nevertheless, mass loss may include disintegration, leaching of soluble fractions, material detachment, and biological activity; by itself, it does not demonstrate mineralization, as emphasized in critical analyses of biodegradable-plastic communication and assessment [29]. ASTM D5988-18(2025) determines aerobic biodegradation in soil through evolved carbon dioxide, whereas ISO 17556:2019 uses oxygen demand in a closed respirometer or the amount of carbon dioxide evolved [30,31]. Because the experiment analyzed here did not include respirometry, reference material, or CO_2_ or O_2_ quantification, its gravimetric responses are termed “apparent mass loss in soil” and “apparent mass loss under outdoor exposure”, without the implication of equivalence to standardized biodegradation measurements.

Previous studies have separately examined starch–Opuntia films [9,10,18], gelatin-containing Opuntia films [11,12,21], starch–gelatin matrices [3,4,5,22], mixture–process optimization [1,2], and gravimetric or biodegradation-related tests [16,19,20,21,22]. The present contribution is not a new polymer chemistry, a newly identified Opuntia species, or the invention of mixture–process optimization. Their specific contribution consists of a comprehensive analysis, within a formulation-specific design, of a variable mixture of starch and gelatine with a constant content of Nopal-*Opuntia* spp. mucilage, including post-drying heat treatment factors, three mechanical responses and two apparent mass loss responses measured separately. A focused search of Scopus and publisher sources through 11 September 2026 identified no directly matching study integrating these particular elements; this search result is not a claim that no related studies exist. The study therefore evaluates and models the joint effects of starch–gelatin ratio, temperature, and time on tensile stress, modulus, elongation, and apparent mass loss in soil and outdoors over 48 days. Individual predictions and multiresponse compromises are reported within the studied domain. Because one film was manufactured per design point and no confirmatory runs were conducted, predictions are exploratory.

## 2. Materials and Methods

### 2.1. Raw Materials and Mucilage Extraction

Fresh cladodes belonging to the genus Opuntia were used, specifically, the mucilage of the nopal (Andean Tuna), an Andean species from Ecuador; the material was designated as *Opuntia* spp. Cladodes showing physical damage, mold, or visible impurities were discarded; the selected cladodes were washed with distilled water and cut into pieces to promote mucilage release. The chopped material was immersed in bottled water for 48 h, after which the mucilaginous phase was separated by filtration through a strainer. The original study records did not retain the cladode-to-water ratio, extraction yield, solids content, pH, or chemical composition. These properties were not recorded in the retained records and cannot be reconstructed from the thesis or reconciled dataset; no estimates have therefore been introduced.

In addition to mucilage, starch, gelatin, glycerol, and vinegar of the types used commercially in Ecuador were used; however, the study records do not identify the manufacturers, batches, grades, starch botanical origin, gelatin bloom strength, glycerol purity, or acetic acid concentration in vinegar. These acquisition and characterization details were not retained in the original documentation. Since they cannot be verified retrospectively, undocumented product specifications have not been inferred. Their absence limits reproducibility and precludes attributing observed differences to material grade or composition-related purposes.

### 2.2. Mixture–Process Design

The experiment combined four starch–gelatin ratios with two post-drying thermal-treatment temperatures and two treatment times, yielding 16 runs. Total starch plus gelatin mass was fixed at 7.182 g, while mucilage (200 mL), glycerol (2.79 g), and vinegar (1.99 g) were held constant. Starch and gelatin fractions were expressed relative to the sum of the two components. Temperature (40 and 60 °C) and treatment time (5 and 10 min) were the process factors. The original matrix was generated as a DOE in Minitab (22.4.0); neither the project file nor software version was retained. Mixture–process designs and related optimization strategies have precedents in biopolymer-film research [1,23,24]; their use here is methodological context and is not presented as a novelty claim.

One film was manufactured for each of the 16 design points, as executed in the thesis study [32]. The original design did not include independently manufactured repeat films under a common composition–process combination. The three tensile specimens and specimens assigned to each exposure condition were cut from that run’s single film; they are technical subsamples, not independent process replicates. The available records do not document a rationale for choosing one film per run, so no post hoc explanation based on cost, resources, or experimental constraints is asserted. No pure manufacturing error or confirmatory lack-of-fit estimate is available.

### 2.3. Film Preparation

For each run, 200 mL of mucilage was measured, and the components were weighed, as described in Table 1. Ingredients were incorporated in the following order: mucilage, glycerol, vinegar, and finally gelatin and starch. The mixture was stirred continuously by hand and heated at a reported range of 70–90 °C until it appeared homogeneous and starch gelatinization was promoted. It was poured into previously conditioned glass molds, dried at room temperature for approximately 1–2 days, and demolded. Each film then received the assigned post-drying thermal treatment in an electric drying oven set to 40 or 60 °C for 5 or 10 min. This physical treatment is not termed chemical curing, because no crosslinking reaction was demonstrated.

The retained records do not specify volume poured per mold, mold dimensions, heating rate, the actual temperature reached by each mixture or film, stirring rate, ambient temperature or humidity during drying, or final film mass. These variables were not documented in the source records and cannot be recovered; they may have contributed to between-run variation, and this omission limits reproducibility. They were not replaced with assumed values. The reported oven settings and broad heating interval are the only available temperature information.

### 2.4. Tensile Testing

Three type IV specimens were cut from each film. The thesis reported an approximate mean film thickness of 0.5 mm. The records identify ASTM D638-14 [33] as the procedure used, and specimens were tested in a Shimadzu AGS-X universal testing machine at a crosshead speed of 5 mm/min. Maximum tensile stress, the modulus reported in the available test sheets, and elongation at break were transcribed from those records. The mean and standard deviation of the three specimens were calculated for each run, and the film mean was used as the DOE response. ASTM D882 is specifically scoped to plastic films and sheeting below 1.0 mm [34]; the reported approximate thickness falls within that scope. The surviving record does not establish full compliance with either standard.

The specimen geometry and ASTM D638-14 designation are reported as stated in the thesis record, not as a claim of demonstrated standard compliance. Individual specimen widths and thicknesses, machine-entered cross-sectional area, native instrument files, complete stress–strain curves, preconditioning, initial grip separation, and the modulus calculation interval were not retained. Consequently, the reported stress values were transcribed from the original test sheets and could not be independently recalculated from verified cross-sectional dimensions. The approximate 0.5 mm thickness makes ASTM D882 [34] relevant for thin-film testing, but the specimens were not retested under that method. The modulus remains provisional, and all three specimens are subsamples of one film.

### 2.5. Apparent Mass-Loss Monitoring

#### 2.5.1. Specimen Preparation and Allocation

Six distinct specimens were cut from each film to a target mass of approximately 2 g; their dimensions and surface areas were not standardized. The retained calculation record used a common nominal initial mass of 2.00 g for every specimen because individual day-0 masses were not preserved. The six specimens were randomly assigned to two exposure conditions: three to soil burial and three to direct outdoor exposure. The randomization sequence was not retained. Specimens assigned to both conditions came from the same film, so the design preserved formulation traceability but did not provide independent manufacturing replicates. Gravimetric soil-biodegradation standards use evolved CO_2_ or oxygen demand, rather than mass disappearance alone [30,31].

Monitoring was conducted in Ambato, Tungurahua, Ecuador, from 2 November to 20 December 2025. The former was defined as day 0 and the latter as day 48. The same specimens were weighed every two days; thus, time points were treated as repeated measurements rather than new independent observations.

#### 2.5.2. Soil-Burial Exposure

Soil was collected on 31 October 2025 from a single location in an agricultural area of Huachi Grande, Ambato. Surface vegetation and litter were removed before sampling, and material was taken from the upper 20 cm of the profile. The soil was mixed manually as collected, without drying or sieving, and distributed to a visually similar height among 16 polyethylene terephthalate containers, one per formulation. Soil mass and volume per container were not determined. The prepared containers remained open and outdoors for the two days preceding day 0.

Each container was approximately 7 cm in diameter and 7 cm deep. Four 2 mm diameter drainage holes were made in the base, and a polypropylene mesh with an approximate 2 × 2 mm opening was placed inside to retain the soil and facilitate drainage. The 16 units were arranged in a fixed 4 × 4 matrix, approximately 5 cm apart, following the DOE run order; the spatial arrangement was not randomized.

The three specimens from each formulation were placed horizontally 5 cm below the surface, fully covered, and not touching each other. After each weighing, each specimen was returned to the same container, position, depth, and orientation, and the soil was replaced without intentional compaction. Containers remained open at the top and exposed to local outdoor conditions. No irrigation was applied; moisture originated from precipitation and ambient exposure. No visible water accumulation was observed, although drainage rate and time were not measured.

Soil moisture was checked with a portable Yieryi YY-1000 m. On day 0, an average of 65% was obtained from three readings at nearby points in the outdoor soil before its distribution among the containers. On days 15 and 48, three readings were taken in each of the 16 containers; the overall means of the 48 readings were 68 and 72%, respectively. Three additional readings were taken at nearby points in the outdoor soil on those dates, and their averages agreed with the values reported for the containers. Only the means were retained, precluding reconstruction of their dispersion. Temperature checks were performed with a thermometer and an approximate range of 15–18 °C was reported, but no measurement log was retained; the range is therefore presented only as contextual information and not as a controlled condition.

#### 2.5.3. Direct Outdoor Exposure

Specimens assigned to outdoor exposure were placed in a single open rectangular polypropylene tray with a base of approximately 55 × 45 cm and a depth of 2 cm. The base contained four 2 mm drainage holes. A polypropylene mesh elevated 0.5 cm by plastic spacers was installed over the base so that both specimen faces remained exposed and water could drain underneath.

The 48 specimens—three from each of the 16 formulations—shared this enclosure and remained horizontal, individually identified, separated, and without contact or overlap. Their positions were randomly assigned at the beginning and remained fixed throughout the test. After each weighing, every specimen was returned to the same position and orientation. The setup was directly exposed to solar radiation, rain, and wind; the tray prevented specimen displacement but did not constitute an independent replicate of the macroenvironment.

#### 2.5.4. Cleaning, Weighing, and Response Calculation

At each measurement time, specimens were removed from their respective setups, cleaned with distilled water at room temperature, and air-dried for 10 min before weighing. They were not dried to constant mass. Measurements used a CAMRY EHA601 balance (Zhongshan Camry Electronic Co., Ltd., Zhongshan, China) with a maximum capacity of 200 g and a nominal resolution of 0.01 g. The researcher reported that the new instrument was factory-calibrated, but no certificate, calibration label, reference weight, uncertainty statement, or metrological documentation was retained. Only nominal resolution is therefore reported; traceable calibration or accuracy is not claimed.

The apparent mass loss of each specimen at time *t* was calculated relative to the common nominal mass:

(1) $$L_{t}\;(\%)\;=\;\frac{2.00\;-\;m_{t}}{2.00}\;\times \;100$$

where m_t_ is the measured mass, in grams, after cleaning and 10 min of air drying. A 0.01 g display increment corresponds to 0.5 percentage points in the calculated response when divided by the nominal 2.00 g denominator; half of one display increment corresponds to ±0.25 percentage points from final-mass rounding alone, conditional on an exact denominator. This is not a total uncertainty interval. The specimen-specific initial masses, residual moisture, cleaning-related material loss, and dimensions were not recorded, so their contributions cannot be quantified. The response is consequently treated as an exploratory apparent-mass-loss index and not as a precise estimate of dry-mass degradation or mineralization.

### 2.6. Data Structure and Units of Analysis

The analytical dataset comprised 16 runs, 48 tensile records (three specimens per film), and 2400 longitudinal mass records (16 films × 2 conditions × 3 specimens × 25 measurement times from day 0 to day 48). The single film was the experimental unit for composition and post-drying thermal-treatment factors. Mechanical DOE responses were the means of three technical specimens per film. The two day-48 gravimetric responses were the means of the three specimens from the same film in each setup, computed with the common nominal 2.00 g denominator. Standard deviations describe within-film or between-specimen dispersion only; they do not quantify manufacturing variation or uncertainty in the nominal starting mass.

Observations within a film, a container, or a temporal trajectory were not treated as independent replicates of manufacturing factors. Complete trajectories were retained for the temporal description of each condition. Because the soil-burial and outdoor-exposure conditions lacked equivalent replicated macroenvironments, they were analyzed as separate responses rather than as replicates of a single environmental factor.

### 2.7. Statistical Modeling and Diagnostics

Reanalysis was performed using the reconciled and approved master dataset, without modifying the original files. Gelatin fraction, *x_G*, was used as the mixture coordinate, with *x_A* = 1 − *x_G*, where *x_A* is the starch fraction. Three hierarchical structures were compared for each of the five responses:

1. M1, main effects:

*y* = *β*_0_ + *β*_1_*x_G* + *β*_2_*T* + *β*_3_*t* + *ε*.

2. M2, quadratic–interaction model:

*y* = *β*_0_ + *β*_1_*x_G* + *β*_2_*x_G*^2^ + *β*_3_*T* + *β*_4_*t* + *β*_5_*Tt* + *β*_6_*x_GT* + *β*_7_*x_Gt* + *ε*.

3. M3, quadratic by process condition: An independent quadratic curve in x_G for each observed temperature–time combination, with 12 parameters.

Models were assessed using *R*^2^, adjusted *R*^2^, root mean square error, corrected Akaike information criterion, and predicted *R*^2^ from leave-one-formulation-out refits. Because only 16 film-level experimental units were available, the cross-validation results are treated as internal diagnostic estimates rather than definitive external validation; small-sample cross-validation can itself be unstable [25,26]. Influence was examined using Cook’s distance, with 4/n = 0.25 as a practical flag, and externally studentized residuals. Influential formulations were not removed automatically. A confirmatory lack-of-fit test against pure manufacturing error was not possible, because no independent replicate films were produced.

M3 was excluded from all prediction and optimization because of overfitting and negative predictive performance. M1 was selected for apparent mass loss in soil on day 48. M2 was retained with exploratory scope for maximum stress, modulus, elongation, and apparent mass loss under outdoor exposure. Selection balanced parsimony, fit, cross-validation, and influence rather than relying on *R*^2^ alone.

### 2.8. Individual and Multiresponse Optimization

Optimization was restricted to the experimental domain: 0 ≤ xG ≤ 0.75, xA = 1 − xG, 40 ≤ T ≤ 60 °C, and 5 ≤ t ≤ 10 min. Temperature and time were treated as continuous quantitative variables only to be interpolated through estimable linear and interaction terms. They were experimentally tested at two levels each, and no quadratic temperature or time terms were estimated; intermediate values therefore do not represent experimentally observed conditions or evidence of curvature. Each of the five responses was maximized individually. For the composite desirability [27], the primary scenario assigned importance 2 to each of the three mechanical responses and importance 1 to each of the two gravimetric responses. Thus, apparent mass loss received half the importance of any one mechanical response. Equal importance for all five responses and a gravimetric-loss-emphasis scenario were retained only as sensitivity analyses because mass loss used a nominal initial denominator and was not measured to constant dry mass.

Each of the five responses was maximized separately during individual optimization. For overall optimization, each prediction *ŷ_i_* was transformed into a linear individual desirability *d_i_*, equal to 0 at the minimum observed value and 1 at the maximum observed value among the 16 film means. Composite desirability was calculated as the weighted geometric mean:

(2) $$D\;=\;{\left(\prod_{i=1}^{5}{d_{i}}^{r_{i}}\right)}^{\frac{1}{\left(\sum_{i=1}^{5}r_{i}\right)}}$$

where r_i_ denotes the importance assigned to each response. Three scenarios were evaluated: balanced, with equal importance for all five responses; mechanical priority, with a 2:1 mechanical-to-gravimetric-loss importance ratio; and moderate gravimetric-loss emphasis, with a 1:2 ratio. Desirability limits were relative to the experimental dataset and were not interpreted as normative or application specifications.

The maximum was located deterministically using an initial grid followed by two successive local refinements. Stability was assessed by refitting models and repeating optimization after omitting one complete formulation at a time. Ratios of gravimetric-loss to mechanical-response importance from 1:1 to 7.5:1 were explored as sensitivity analyses. In every case, the continuous predicted maximum was distinguished from the best actually observed run. No independent film was manufactured under a predicted continuous condition; all predicted solutions therefore remain exploratory and unconfirmed.

### 2.9. Generative Artificial Intelligence-Assisted Workflow

Perplexity Pro—Claude Opus 5, Perplexity AI, San Francisco, CA, USA accessed in September 2026—was used to identify candidate papers in the scientific literature. OpenAI Codex—GPT-6 Astra, OpenAI, San Francisco, CA, USA, accessed in September 2026—supported document organization, the development of scripts for data verification, part of the statistical analysis, and visualization. No generative AI tool produced or modified the primary experimental data. The authors verified the bibliographic records against the original publications, checked and analyzed the numerical results against the master dataset, critically reviewed all scientific interpretations, and drafted the complete manuscript and figures. The authors assume full responsibility for the originality, validity, and integrity of the published content.

### 2.10. Experimental Data Provenance and Reanalysis

The primary experiments were performed by G.R.F.C. as part of an Engineering thesis deposited in the repository of the Technical University of Ambato [32]. The present article provides a more detailed analysis of the results of the experimentation, considering biomaterial in terms of tensile stress, and weight loss as biodegradation. It uses the measurements generated in the thesis and the preserved test sheets, together with a master dataset reconciled and reviewed by the authors; gravimetric analysis was limited to days 0 to 48. Bibliographic synthesis, data reconciliation, model comparison using the “leave out a formulation” method, influence diagnosis, continuous individual and multiple response optimization, sensitivity analyses, and the interpretation presented here were recently performed or composed for this manuscript. The original acquisition records, individual initial masses, traction machine files, and some procedural details were not reconstructed. The thesis is cited as the source of the experimental study so that the origin and scope of the reanalysis are transparent.

## 3. Results

### 3.1. Observed Mechanical Responses

Mechanical properties varied among the 16 composition–process combinations (Table 2). Mean maximum tensile stress ranged from 0.534 MPa for F10 to 1.614 MPa for F04. F04—starch/gelatin 0.25/0.75, 40 °C, and 5 min—showed the highest recorded stress, followed by F08 and F12 at 1.305 and 1.204 MPa, respectively. These three runs shared the highest gelatin fraction, although their post-drying thermal treatments differed. The values are descriptive because verified specimen-specific cross-sectional areas and raw stress–strain records were unavailable.

**Figure 1:**
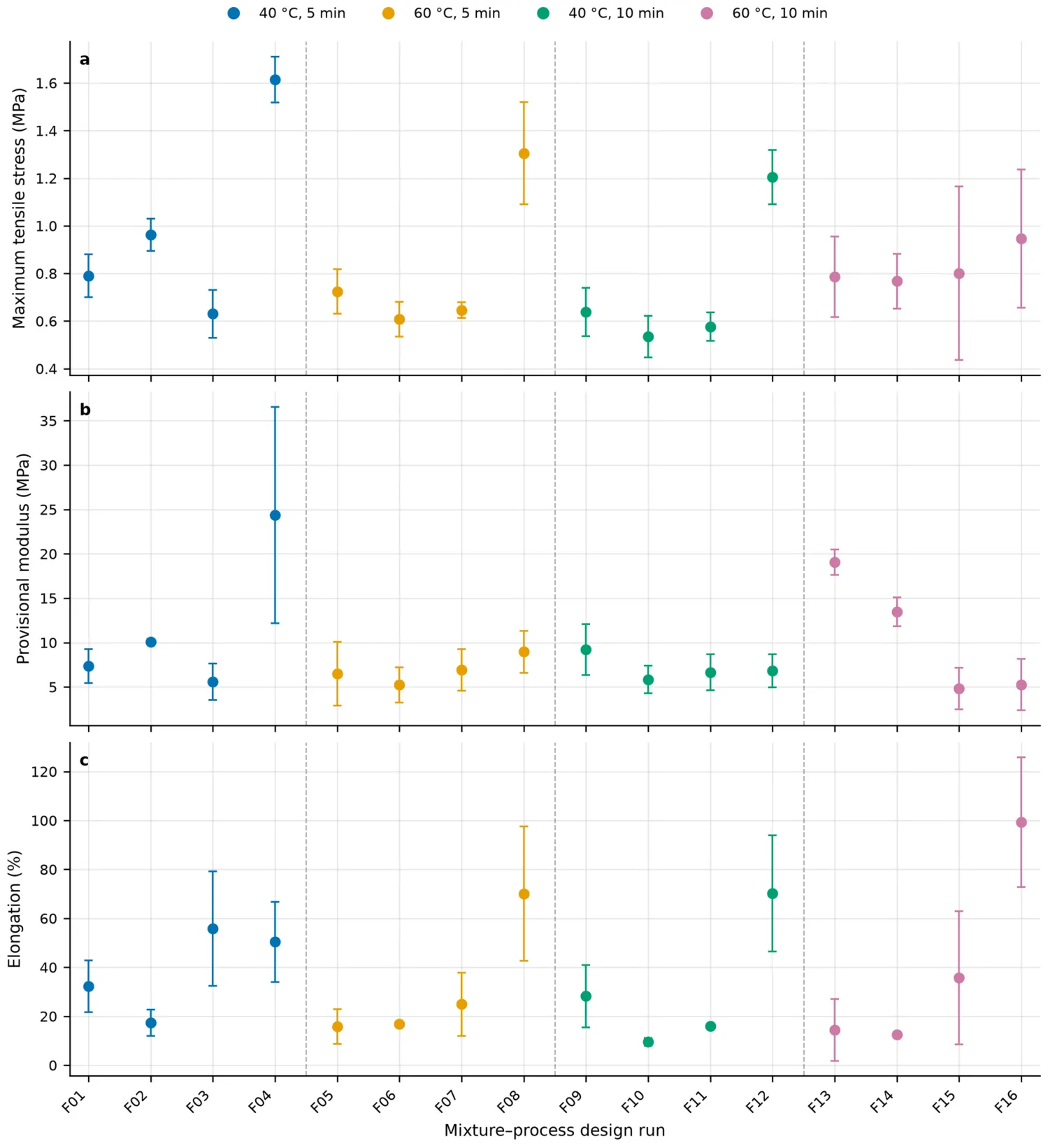
Observed mechanical properties of films obtained through the mixture–process design: (**a**) maximum tensile stress; (**b**) provisional modulus; and (**c**) elongation. Points represent the means and bars the standard deviations of the three technical specimens cut from one film per run. Colors identify the four post-drying thermal-treatment combinations. Error bars do not represent variation among independent film-manufacturing replicates, and stress values could not be recalculated from specimen-specific cross-sectional measurements.

The mean provisional modulus ranged from 4.813 MPa for F15 to 24.358 MPa for F04. F13 and F14 ranked next, at 19.070 and 13.463 MPa. Within-film specimen dispersion was particularly high for F04 (24.358 ± 12.176 MPa), whereas F13 and F14 showed smaller dispersions, as shown in Figure 1. Elongation ranged from 9.564% for F10 to 99.269% for F16. The deviations among the three specimens from F16, F12, and F08 indicate within-film technical heterogeneity. These standard deviations are not the independent manufacturing-replicate uncertainty, and the modulus is provisional because the modulus interval and verified specimen dimensions are unknown.

Descriptive comparison of the four post-drying thermal-treatment settings showed that the highest gelatin fraction produced the greatest recorded stress at 40 °C and 5 min, but did not simultaneously retain the highest provisional modulus and elongation across treatments. F04 had the highest recorded stress and modulus, whereas F16—the same mixture ratio treated at 60 °C for 10 min—had the highest elongation. Because these observations come from one film per design point, they motivate model-based exploration but do not establish manufacturing repeatability.

### 3.2. Apparent Mass-Loss Evolution

Apparent mass loss increased over 48 days under both conditions. As a descriptive summary of the 16 formulation means, soil burial reached 14.59 ± 3.08% on day 8, 36.53 ± 6.04% on day 16, 54.03 ± 6.37% on day 24, and 80.58 ± 6.98% on day 48. Corresponding outdoor-exposure values were 8.28 ± 1.49, 21.03 ± 3.58, 33.38 ± 4.87, and 76.78 ± 8.64%. These statistics summarize the distribution among formulations and do not represent independent environmental replicates. This is detailed in Table 3 below.

**Table 3:** Descriptive evolution of apparent mass loss. Values correspond to the mean ± standard deviation among the 16 formulation means.

Day	Soil Burial (%)	Range Among Formulations (%)	Outdoor Exposure (%)	Range Among Formulations (%)
0	0.00 ± 0.00	0.00–0.00	0.00 ± 0.00	0.00–0.00
8	14.59 ± 3.08	7.50–20.00	8.28 ± 1.49	5.50–10.00
16	36.53 ± 6.04	25.50–51.00	21.03 ± 3.58	16.00–29.50
24	54.03 ± 6.37	46.50–67.50	33.38 ± 4.87	24.50–42.50
32	65.28 ± 6.71	56.00–77.00	48.09 ± 6.71	39.00–60.00
40	73.16 ± 6.95	62.00–85.00	62.69 ± 7.99	52.00–80.50
48	80.58 ± 6.98	69.50–93.00	76.78 ± 8.64	65.50–95.00

**Figure 2:**
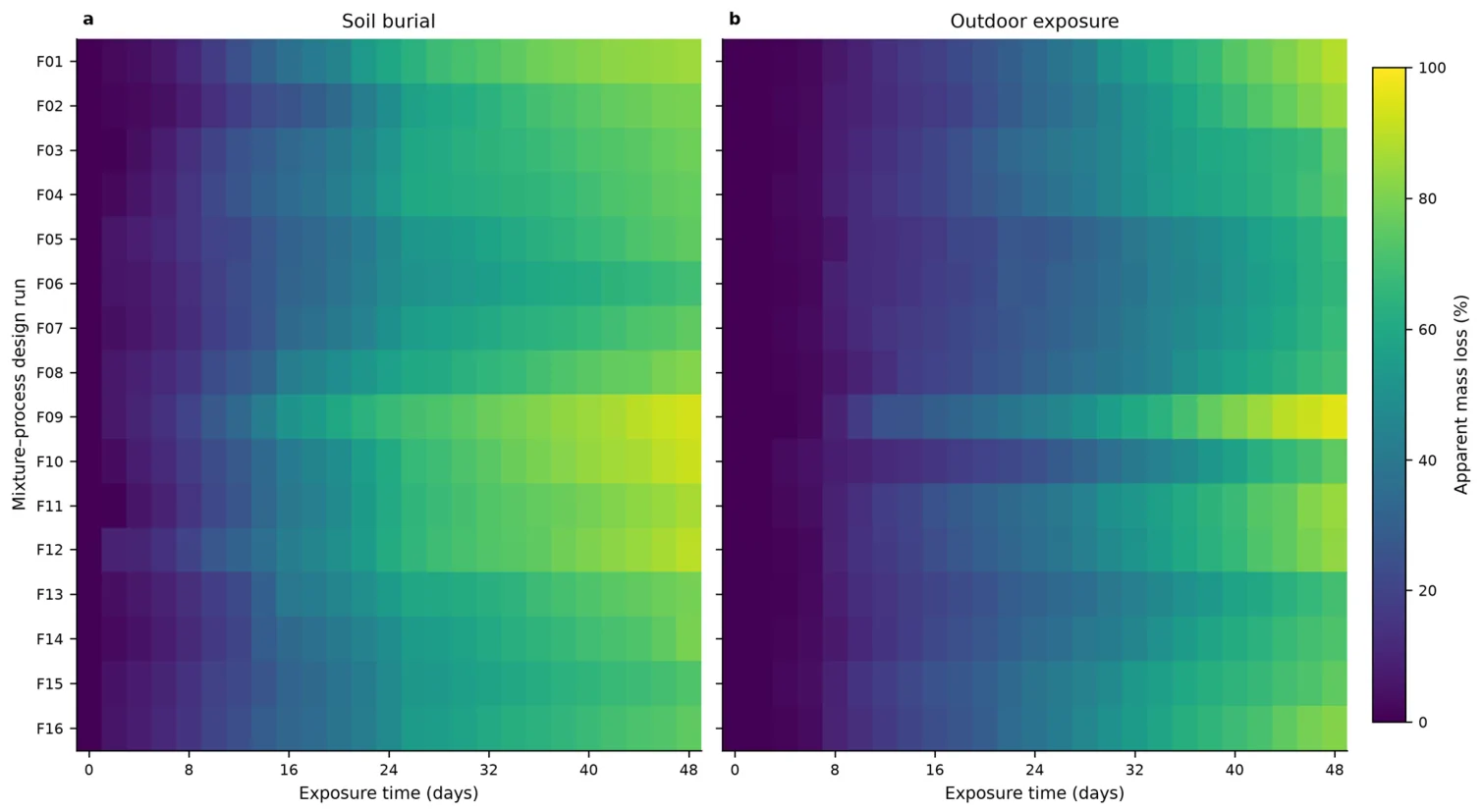
Evolution of apparent mass loss over 48 days. Curves show means of three technical specimens per formulation and measurement times under (**a**) soil burial and (**b**) outdoor exposure. The common color scale supports descriptive comparison. Each setup was conducted in a single macroenvironment; the figure supports neither an inferential comparison between environments nor a claim of ultimate aerobic biodegradation. Values use a common nominal starting mass and do not include unquantifiable initial-mass or residual-moisture uncertainty.

On day 48, the calculated formulation means ranged from 69.5 to 93.0% in soil and from 65.5 to 95.0% outdoors. F09—starch/gelatin 1.00/0.00, 40 °C, and 10 min—had the largest calculated apparent losses under both conditions: 93.0 ± 0.5% in soil and 95.0 ± 0.5% outdoors, as shown in Figure 2. These percentages are retained as descriptive outputs of the recorded calculation, not as metrologically complete degradation estimates: the initial masses were nominal, dimensions were not standardized, and specimens were not dried to constant mass. The 0.5 percentage-point increments reflect the 0.01 g balance display relative to a 2.00 g denominator.

The 96 individual trajectories were predominantly decreasing in mass. Three increases between consecutive measurements occurred among the 48 soil trajectories and six among the 48 outdoor trajectories. The largest point increase was 0.03 g in soil and 0.01 g outdoors. These values were retained without smoothing or correction, because they belonged to the original records and were close to the instrument resolution of 0.01 g.

### 3.3. Model Selection and Predictive Performance

The 12-parameter M3 model produced *R*^2^ values from 0.861 to 0.959, but negative predicted *R*^2^ values for all five responses, ranging from −5.190 to −0.801, together with a maximum leverage of 0.95. This behavior indicated overfitting and led to its exclusion from prediction and optimization. Table 4 summarizes the performance of the selected models.

**Figure 3:**
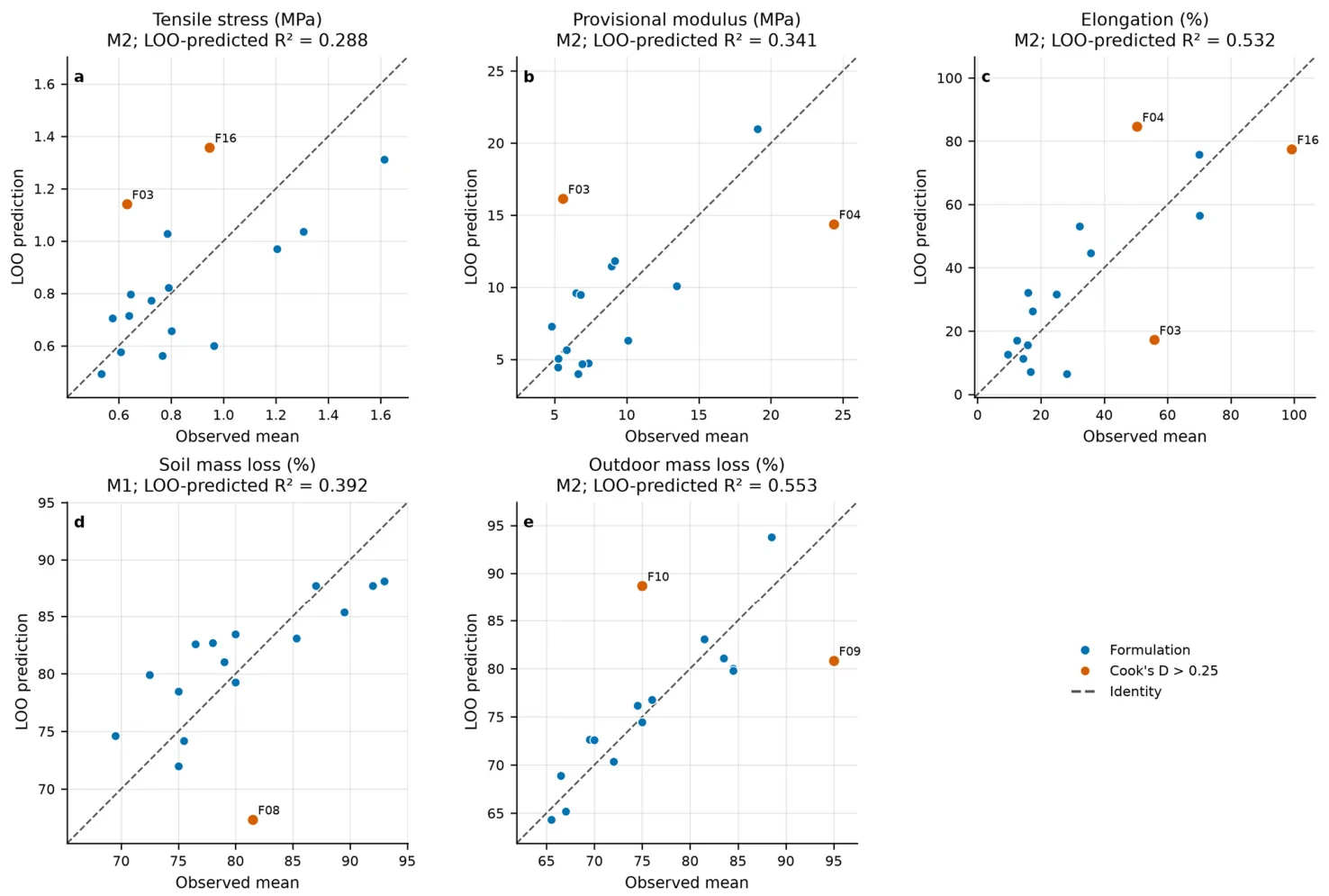
Cross-validation of the selected models by leaving one complete formulation out of each refit: (**a**) maximum stress, M2; (**b**) provisional modulus, M2; (**c**) elongation, M2; (**d**) apparent soil mass loss on day 48, M1; and (**e**) apparent outdoor mass loss on day 48, M2. The dashed line denotes identity between observation and prediction. Orange points identify formulations with Cook’s distance greater than 0.25 in the full fit; they were not removed. Predicted *R*^2^ was calculated using the mean of each film as the unit of analysis.

Among the selected models, LOO-predicted *R*^2^ was 0.532 for elongation and 0.553 for outdoor apparent mass loss. Stress and provisional modulus had the lower values of 0.288 and 0.341, indicating limited predictive performance. For soil apparent mass loss, M1 and M2 yielded nearly identical predicted *R*^2^ values (0.392 and 0.394); M1 was retained because it used four rather than eight parameters, as show in Figure 3. These diagnostics apply to the 16 film means and do not overcome the absence of independent film replicates.

Influence analysis identified Cook’s distances greater than 0.25 for F03 and F16 in the stress model; F03 and F04 in the modulus model; F03, F04, and F16 in the elongation model; F08 in the soil-loss model; and F09 and F10 in the outdoor-loss model. These observations were retained in the fits. Their effect was assessed by successively omitting one complete formulation and was carried forward into the stability analysis of predicted optima.

### 3.4. Individual Optimization

Predicted maxima for each response occurred at boundaries of the experimental region (Table 5). The stress model predicted 1.508 MPa for starch/gelatin 0.25/0.75, 40 °C, and 5 min, whereas the highest observed value was 1.614 MPa for F04 under the same combination. The modulus had the same predicted location, with 20.866 MPa compared with 24.358 MPa observed for F04. For elongation, the solution shifted to 60 °C and 10 min, with 91.626% predicted and 99.269% observed for F16.

Both gravimetric responses favored the gelatin-free formulation. The predicted maximum soil mass loss was 89.583% at starch/gelatin 1.00/0.00, 40 °C, and 10 min. For outdoor exposure, the model predicted 90.350% with the same mixture and temperature but at 5 min. F09 was the best observed run for both responses and reached 93.0 and 95.0%, respectively, at 10 min.

The locations of maximum stress, elongation, and soil mass loss were unchanged across the 16 leave-one-formulation-out refits. In the outdoor-loss model, gelatin fraction 0 and temperature 40 °C were retained in all 16 refits, but time alternated between 5 and 10 min. The location of maximum modulus was sensitive to the omitted formulation and shifted among several domain boundaries; it was therefore not considered robust.

### 3.5. Overall Optimization and Sensitivity Analysis

Simultaneous optimization confirmed that no single condition maximized all five responses. Under the primary mechanically dominant weighting (importance 2 for each mechanical response and 1 for each gravimetric response), the modeled compromise was starch/gelatin 0.25/0.75, 40 °C, and 5.00 min (D = 0.64388), matching the conditions of observed run F04. Table 6 shows that, with equal importance for every response, the sensitivity solution was 0.25/0.75, 40 °C, and approximately 5.4 min (D = 0.58455). Under gravimetric-loss emphasis (importance ratio 1:2, mechanical:gravimetric per response), the sensitivity solution remained 0.25/0.75 at 40 °C and approximately 7.7 min (D = 0.55349). The latter two times are interpolated model predictions, not tested conditions.

**Table 6:** Solutions and predicted responses for multi-response optimization scenarios.

Scenario	Starch	Gelatin	Temperature (°C)	Time (min)	Stress (MPa)	Modulus (MPa)	Elongation (%)	Soil Loss (%)	Outdoor Loss (%)	*D*
Balanced 1:1	0.250	0.750	40.0	5.44	1.474	19.711	62.611	81.263	75.753	0.58455
Mechanical priority 2:1	0.250	0.750	40.0	5.00	1.508	20.866	62.343	80.750	75.088	0.64388
Mass loss 1:2	0.250	0.750	40.0	7.71	1.299	13.761	63.993	83.909	79.183	0.55349

For the equal-importance sensitivity scenario, F04 was the best observed run and had D = 0.52879, below the interpolated prediction at approximately 5.4 min. Under the primary mechanically dominant weighting, the modeled solution coincided with F04; its desirability calculated from observed responses was 0.60855, compared with 0.64388 from fitted model predictions. Under gravimetric-loss emphasis, F12 was the best observed run (D = 0.52908), while the continuous sensitivity solution at approximately 7.7 min had predicted D = 0.55349. None of these comparisons constitutes independent confirmation.

Omitting one formulation retained a gelatin fraction of 0.75 and a temperature of 40 °C in all 16 refits for the three weighting scenarios. Time was less stable: approximately 5.0–6.1 min under equal response importance, 5.0–5.4 min under the primary mechanically dominant weighting, and 6.6–8.1 min under gravimetric-loss emphasis. These ranges describe sensitivity to omitting one formulation; they are not confidence intervals and do not quantify manufacturing uncertainty.

**Figure 4:**
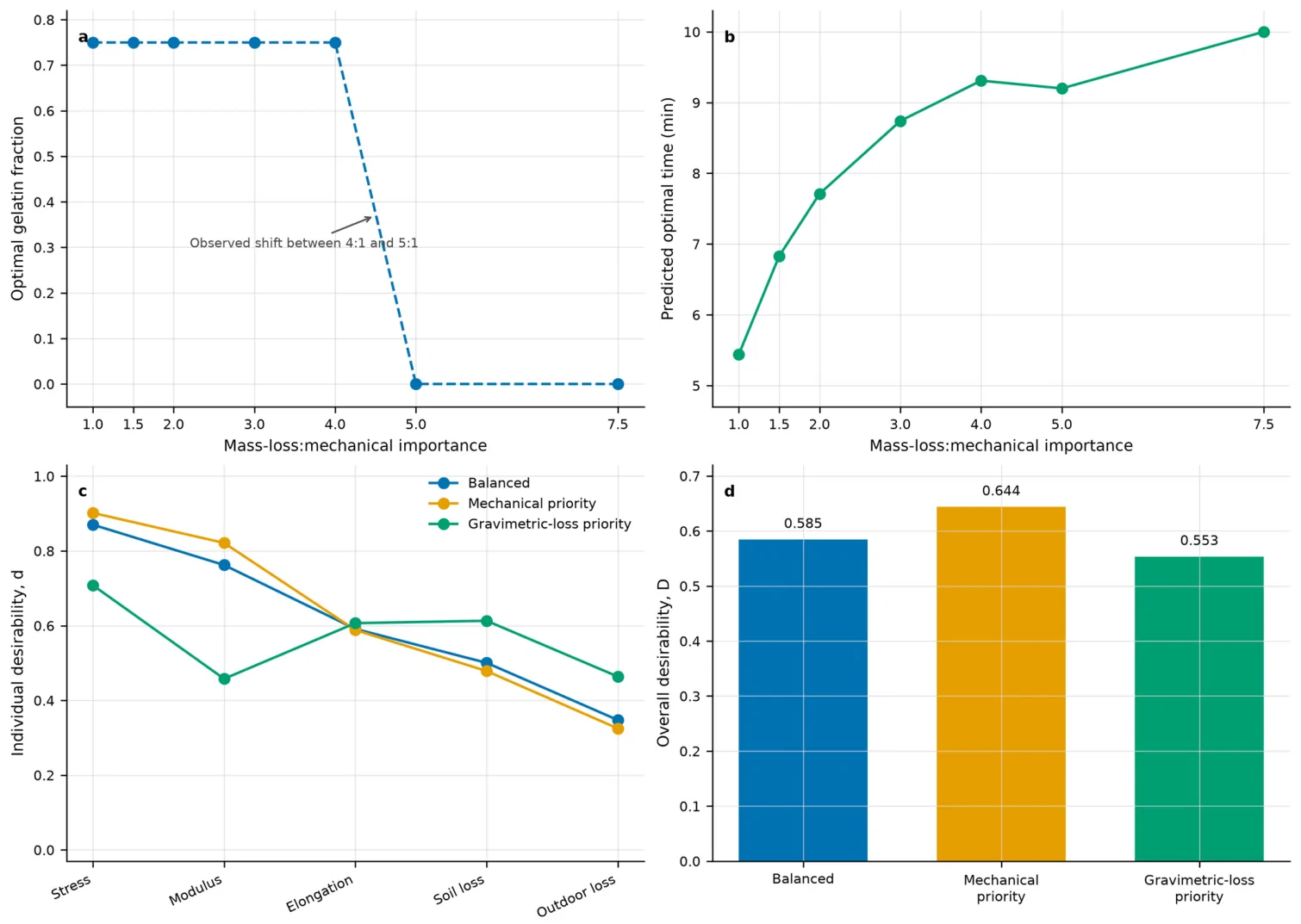
Multiresponse optimization and sensitivity to gravimetric-loss importance. (**a**) Gelatin fraction and (**b**) treatment time of the modeled solution as the gravimetric-loss-to-mechanical importance ratio increased; (**c**) individual desirabilities for five responses under equal, mechanically dominant, and gravimetric-loss-emphasis scenarios; and (**d**) overall desirability. The plotted intermediate values are within-domain predictions from models fitted at two treatment-time levels. The mixture shift is bounded between ratios 4:1 and 5:1; no exact continuous threshold was estimated. D values across different importance settings are not directly comparable. All solutions are unconfirmed model predictions.

As gravimetric-loss importance was progressively increased, the solution retained starch/gelatin 0.25/0.75 and 40 °C through a gravimetric-to-mechanical importance ratio of 4:1, while time increased from approximately 5.4 to 9.3 min. Between ratios 4:1 and 5:1, the modeled mixture shifted to pure starch. At 5:1 the predicted time was approximately 9.2 min; at ratios of 7.5:1 or greater it reached 10 min and the modeled combination coincided with F09. This is a sensitivity to analyst-assigned desirability weights, not a physical threshold or evidence of an environmentally superior material. Given the limitations of the gravimetric response, this high-weight region is not used as the principal conclusion, as show in Figure 4.

### 3.6. Scope of the Modeled Results

The models describe the 16 manufactured films and generate interpolations within the experimental region. Cross-validation and influence diagnostics showed unequal predictive performance, particularly for stress and provisional modulus. The standard deviations in Table 2 describe three specimens cut from one film and do not estimate variation among independently manufactured films. The gravimetric responses additionally rely on a common nominal initial mass and short air drying; no total uncertainty interval can be recovered. Intermediate time values were not tested. Model predictions and desirability solutions are therefore exploratory, formulation-specific, and presented alongside observed runs.

## 4. Discussion

### 4.1. Trade-Off Among Composition, Processing, and Mechanical Response

Variation in the three mechanical responses confirms that the starch–gelatin ratio cannot be interpreted separately from post-drying thermal-treatment conditions. F04, F08, and F12, which had the greatest recorded stress, contained the highest gelatin fraction, but the maximum occurred at 40 °C for 5 min. At the same composition, F16 treated at 60 °C for 10 min had the greatest elongation but not the highest recorded stress or provisional modulus. Related starch–gelatin studies also report composition-dependent mechanical responses [3,4,5,22], while Opuntia mucilage–gelatin films have been assessed for tensile behavior [12]. These studies support comparison of response patterns, not direct transfer of numerical properties between formulations.

This result is consistent with the system-specific nature of film-forming matrices. Todhanakasem et al. [12] measured stress, modulus, and elongation in *O. ficus*-indica mucilage–gelatin films; Monteiro et al. [18] evaluated fermented *O. cochenillifera* mucilage with corn starch; and Arias Saldaña et al. [22] examined starch–gelatin films reinforced with Laponite^®^. Niu et al. [3] and Castro et al. [4] likewise studied potato-starch/gelatin and starch/chicken-gelatin matrices. Across these distinct systems, component ratio and processing are relevant, but numerical values cannot be directly compared, because species, mucilage treatment, matrix, proportions, plasticizer, geometry, conditioning, and tensile method differ. The comparison supports a formulation-specific interpretation rather than a general rule that gelatin always improves a given property.

The F04–F16 contrast is compatible with a change in the balance between matrix cohesion and mobility across post-drying thermal treatments, but it does not identify a molecular mechanism. Equilibrium moisture, mucilage solids content, crystallinity, chemical interactions, microstructure, and thermal properties were not determined. The poured volume, mold dimensions, actual film thickness, and individual specimen thicknesses were also unavailable. Claims about crosslinking, compatibility, or thermal degradation would therefore remain hypotheses. Further structural, thermal, microscopic, and moisture-controlled measurements would be needed to test them.

The high within-film dispersion of the modulus in F04 and of elongation in F08, F12, and F16 limits interpretation of the recorded maxima. The three specimens came from one film in each run; dispersion represents subsample heterogeneity and test variability, not between-batch repeatability. The modulus is provisional because its calculation interval and the machine-entered dimensions are unavailable. The thesis identified ASTM D638-14 specimens [33], whereas ASTM D882 is specifically scoped to plastic films and sheeting below 1.0 mm [34]. Since the reported approximate film thickness was 0.5 mm, D882 is the more directly relevant standard to consider in future testing. The surviving records do not permit verification of full compliance with D638 or recalculation of stress from verified cross-sectional area.

### 4.2. Mass Loss Under Two Exposure Conditions

Both setups showed increasing calculated apparent mass loss over 48 days, although their descriptive trajectories differed. At day 48, mean formulation values were 80.58% for soil and 76.78% outdoors; these are not independently replicated environmental means. F09 had the largest recorded gravimetric response in both setups. This pattern is consistent with the relative mass-loss response within these particular containers and tray, but it does not establish environmental performance, biodegradation, or generalizable differences between soil and outdoor exposure.

Previous Opuntia studies provide context but do not establish equivalence. Mannai et al. [19,20] assessed mucilage films blended with styrene–butadiene latex or PVOH and used soil or seawater exposure; Teshager et al. [16] reported gravimetric change after soil burial of mucilage–kaolin films; León-López et al. [21] studied gelatin films containing Opuntia fruit products; and Monteiro et al. [18] evaluated starch films containing fermented O. cochenillifera mucilage. These systems differ in formulation, geometry, exposure, retrieval, washing, drying, and endpoint. Accordingly, their values cannot be pooled or interpreted as comparable rates. The present data add a 48-day, two-setup descriptive series linked to one composition–process matrix, not a standardized degradation rate.

The different trajectories are compatible with multiple possible influences, including moisture, particle contact, leaching, biological activity, solar radiation, rain, wind, washing, and surface erosion; the study did not separate these mechanisms. Each formulation had one soil container in one fixed 4 × 4 spatial arrangement, and all outdoor specimens shared one tray. Thus, the observed differences among formulations describe this single layout and do not estimate environmental-treatment effects or support generalization to other locations, seasons, or soils. This limitation is more consequential than the number of specimens cut from each film, because those subsamples shared the same film and macroenvironment.

The term “apparent mass loss” is essential. Specimens were washed and air-dried for 10 min rather than dried to constant mass, and their individual initial masses were not retained. At a nominal 2.00 g denominator, the balance resolution of 0.01 g corresponds to 0.5 percentage points per display increment; the half-increment rounding contribution is ±0.25 percentage points if the denominator is exact. That instrument-only contribution does not include the unknown deviation of each true initial mass from 2.00 g, residual moisture, cleaning-related loss, or material detachment. Because those quantities were not measured, a complete quantitative uncertainty interval cannot be calculated. Small increases between measurements (up to 0.03 g in soil and 0.01 g outdoors) were retained as observed and may reflect moisture, adhering material, or scale resolution. Gravimetric decrease may include dissolution, leaching, fragmentation, washing, or detachment without mineralization. ASTM D5988-18(2025) measures evolved CO_2_ [30], and ISO 17556:2019 uses oxygen demand or produced CO_2_ [31]; neither endpoint was measured here.

### 4.3. Performance and Limitations of the Mixture–Process Models

Model comparison showed why apparent fit is insufficient for selecting a response surface. M3 achieved high *R*^2^ values, but its predicted *R*^2^ values were negative for all five responses and its maximum leverage reached 0.95. Excluding M3 prevented optimization from relying on a flexible surface that reproduced the available runs but failed when predicting an omitted formulation. This decision is methodologically consistent with the purpose of DOE: to describe trends within the domain without confusing interpolation with additional evidence.

M2 had higher LOO-predicted *R*^2^ than M1 for stress, modulus, elongation, and outdoor apparent mass loss, but used eight parameters for only 16 film-level units. For stress and modulus, predicted *R*^2^ values of 0.288 and 0.341 indicate limited predictive performance. M2 is therefore retained only as an exploratory response surface, not a reliable forecasting model. For soil apparent mass loss, M2 only improved predicted *R*^2^ from 0.392 to 0.394; the more parsimonious M1 was preferred. The use of fit, cross-validation, and model complexity together follows the comparative strategy used in DOE-based film studies [1,23,24], but it cannot substitute for new confirmatory data. Small-sample LOO results are themselves diagnostic and potentially unstable [25,26].

Influential formulations further support this caution. F03 and F16 influenced the stress model; F03 and F04, the modulus model; F03, F04, and F16, the elongation model; F08, the soil-loss model; and F09 and F10, the outdoor-loss model. None was removed, because no measurement error had been documented to justify exclusion. Successively omitting one film showed which components of the solutions remained stable and which depended on observations. This validation respected the actual experimental unit; treating the 48 tensile specimens as independent observations would have artificially inflated the degrees of freedom.

### 4.4. Individual Optima and Overall Compromise

Individual optimization confirmed the partial incompatibility among the objectives. Stress and modulus favored starch/gelatin 0.25/0.75, 40 °C, and 5 min; elongation retained this mixture but shifted to 60 °C and 10 min. The gravimetric responses favored the gelatin-free film at 40 °C. All maxima occurred at domain boundaries. Statistically, these points represent the best predicted directions within the studied range; they do not demonstrate a stationary maximum or justify extrapolation to gelatin fractions above 0.75, temperatures below 40 °C, or times longer than 10 min.

The desirability function formalized the trade-off among the five responses [27]. Under the primary mechanically dominant weighting, the solution matched F04 at 0.25/0.75 starch/gelatin, 40 °C, and 5 min. The equal-weight solution was approximately 5.4 min, while a gravimetric-loss-emphasis sensitivity solution was approximately 7.7 min. Both intermediate values arise from equations fitted to only two treatment-time levels; they neither establish time curvature nor substitute for a tested run. The loss-focused solution should be read as a mathematical sensitivity to analyst-selected weights, not as an environmental recommendation. Leave-one-formulation-out fits retained mixture and temperature but showed appreciable time variation; the modulus also remained sensitive to F04.

The overall solution depends on analytical choices. Desirabilities were scaled between the observed minima and maxima, which are dataset-relative limits rather than performance specifications. The primary analysis now gives twice the importance to each mechanical response as compared to either gravimetric response. Equal response importance and gravimetric-loss emphasis are shown only to make the dependence on weights transparent. D values from different importance settings do not establish that one scenario is intrinsically superior. The mixture shift observed between gravimetric-to-mechanical ratios of 4:1 and 5:1 is a property of this fitted desirability calculation, not a material transition or universal threshold.

Three levels of evidence should be distinguished. F04 is the best observed formulation for the mechanically dominant scenario and matches its modeled condition. F12 is the best observed run under the gravimetric-loss-emphasis sensitivity setting, whereas the corresponding continuous solution near 7.7 min was not tested. F09 is the observed gravimetric-loss extreme. It does not represent a general environmental optimum, and it is favored by the composite calculation only when gravimetric loss receives high analyst-assigned importance. All rankings are conditional on this dataset and the limitations of its response measurements.

### 4.5. Scope, Limitations, and Next Experimental Validation

The study uses a common design to relate formulation, post-drying thermal treatment, mechanical responses, and apparent mass loss, with model comparison, cross-validation, influence diagnostics, and sensitivity analysis. The evidence remains exploratory. One film was produced per combination, independent manufacturing replicates were not utilized, and no confirmatory runs at a predicted continuous condition were performed. Pure manufacturing error and confirmatory lack of fit cannot be estimated. Standard deviations among technical specimens do not replace independent film replication.

Several missing records limit reproducibility: species-level Opuntia identification; mucilage yield, solids, pH, and composition; commercial specifications for the starch, gelatin, glycerol, and vinegar; casting volume and mold dimensions; actual temperature histories; ambient drying conditions; specimen-specific thicknesses; and tensile preconditioning and calculation settings. Gravimetric limitations include a common nominal initial mass, unstandardized specimen dimensions, no constant-mass drying, no controls, a single container per formulation in a fixed spatial arrangement, one shared outdoor tray, and incomplete environmental monitoring. Their effects cannot be reconstructed from the retained data. These limits narrow the claims but do not change the fact that the recorded values are reported as documented.

A subsequent confirmatory study should manufacture independently replicated films at the primary mechanically dominant condition and selected observed comparators, incorporating randomized processing order, measured thickness and area, controlled conditioning, and complete tensile curves. Gravimetric work should record each specimen’s initial mass and dimensions, dry to constant mass under a defined protocol, include blanks and reference materials, and replicate the environmental units and spatial positions. If the objective is ultimate aerobic biodegradation, a respirometric protocol should quantify CO_2_ evolution or oxygen demand with controls under the applicable standard [30,31]. Until then, formulations are predicted or observed candidates and the present environmental endpoint remains apparent mass loss.

## 5. Conclusions

The mixture–process design enabled joint analysis of the starch–gelatin ratio and post-drying thermal-treatment conditions for films containing constant amounts of *Opuntia* spp. mucilage, glycerol, and vinegar. The responses did not reach their maxima simultaneously. F04 had the highest recorded stress and provisional modulus, F16 the highest elongation, and F09 the greatest apparent mass loss in both setups. These findings are descriptive and formulation-specific because each DOE point was represented by one manufactured film.

Run F04—starch/gelatin 0.25/0.75, 40 °C, and 5 min—had the highest recorded maximum stress and provisional modulus, at 1.614 and 24.358 MPa. F16, with the same mixture but a 60 °C, 10 min treatment, had the greatest elongation (99.269%). The stress values could not be independently reconstructed from specimen-specific cross-sectional areas, and no between-film manufacturing variability is available. The results therefore identify observed responses in the retained samples rather than reproducible material properties.

Calculated apparent mass loss increased in both setups over 48 days. F09—starch/gelatin 1.00/0.00, 40 °C, and 10 min—had the largest recorded values (93.0% in soil and 95.0% outdoors) under the common nominal 2.00 g calculation. Because initial masses were not retained, specimen dimensions were not standardized, and samples were not dried to constant mass; the percentages are not precise estimates of biodegradation. No CO_2_ evolution, oxygen demand, or mineralization was measured; ASTM D5988 and ISO 17556 compliance is not claimed.

The model audit showed that a high *R*^2^ does not guarantee predictive performance. M3 was excluded because its LOO-predicted *R*^2^ was negative for all five responses. M1 and M2 were retained for exploratory description, but predictive performance was limited for stress and provisional modulus, and the latter was sensitive to F04. No selected model has independent experimental confirmation. Model-derived surfaces should be restricted to the investigated domain and interpreted as exploratory.

Individual model-based maxima occurred at domain boundaries and indicated different response directions: mechanical properties favored the highest studied gelatin fraction, whereas the two gravimetric responses favored gelatin-free films. For the primary mechanically dominant multiresponse scenario, the model predicted the observed F04 condition—starch/gelatin 0.25/0.75, 40 °C, and 5 min—with D = 0.64388. Equal-weight and gravimetric-loss-emphasis sensitivity scenarios predicted approximately 5.4 and 7.7 min, respectively. These intermediate times were not tested, and no scenario is an experimentally validated optimum. The gravimetric response weights and measured endpoint limitations preclude interpreting the loss-focused scenario as environmental performance.

Overall, F04 is both the observed reference and the model-predicted condition for the primary mechanically dominant scenario. F12 was the best observed run under the gravimetric-loss-emphasis sensitivity setting, while F09 was the observed apparent-mass-loss extreme. Intermediate times remain untested predictions. Before a condition is recommended for an application, independent film-manufacturing confirmation, measured specimen dimensions, complete tensile records, material characterization, and functional-property evaluation are required. A biodegradation claim would require standardized respirometry with controls and reference material.

## Figures and Tables

**Table 1:** Mixing-process design and masses of variable components.

Run	Starch Fraction	Gelatin Fraction	Starch (g)	Gelatin (g)	Temperature (°C)	Time (min)
F01	1.00	0.00	7.1820	0.0000	40	5
F02	0.75	0.25	5.3865	1.7955	40	5
F03	0.50	0.50	3.5910	3.5910	40	5
F04	0.25	0.75	1.7955	5.3865	40	5
F05	1.00	0.00	7.1820	0.0000	60	5
F06	0.75	0.25	5.3865	1.7955	60	5
F07	0.50	0.50	3.5910	3.5910	60	5
F08	0.25	0.75	1.7955	5.3865	60	5
F09	1.00	0.00	7.1820	0.0000	40	10
F10	0.75	0.25	5.3865	1.7955	40	10
F11	0.50	0.50	3.5910	3.5910	40	10
F12	0.25	0.75	1.7955	5.3865	40	10
F13	1.00	0.00	7.1820	0.0000	60	10
F14	0.75	0.25	5.3865	1.7955	60	10
F15	0.50	0.50	3.5910	3.5910	60	10
F16	0.25	0.75	1.7955	5.3865	60	10

**Table 2:** Observed responses by run. Mechanical values are the mean ± standard deviation of three technical specimens cut from one film. Gravimetric values use a common nominal initial mass of 2.00 g; their dispersion does not include uncertainty in that denominator or residual moisture.

Run	Maximum Tensile Strength (MPa)	Provisional Modulus (MPa)	Elongation (%)	Mass Loss (Soil), Day 48 (%)	Mass Loss (Outdoors), Day 48 (%)
F01	0.790 ± 0.090	7.35 ± 1.93	32.23 ± 10.53	85.3 ± 0.3	88.5 ± 0.5
F02	0.963 ± 0.068	10.09 ± 0.31	17.33 ± 5.39	80.0 ± 0.5	84.5 ± 0.5
F03	0.630 ± 0.101	5.58 ± 2.06	55.79 ± 23.45	78.0 ± 0.5	76.0 ± 0.5
F04	1.614 ± 0.097	24.36 ± 12.18	50.38 ± 16.32	76.5 ± 0.5	74.5 ± 0.5
F05	0.724 ± 0.093	6.49 ± 3.59	15.84 ± 7.13	75.5 ± 0.5	66.5 ± 0.5
F06	0.608 ± 0.073	5.23 ± 1.97	16.74 ± 0.98	69.5 ± 0.5	65.5 ± 0.5
F07	0.646 ± 0.033	6.91 ± 2.34	24.89 ± 12.94	75.0 ± 0.5	67.0 ± 0.5
F08	1.305 ± 0.214	8.96 ± 2.36	70.07 ± 27.51	81.5 ± 0.5	69.5 ± 0.5
F09	0.638 ± 0.101	9.19 ± 2.87	28.18 ± 12.75	93.0 ± 0.5	95.0 ± 0.5
F10	0.534 ± 0.087	5.84 ± 1.53	9.56 ± 1.55	92.0 ± 0.5	75.0 ± 0.5
F11	0.577 ± 0.060	6.65 ± 2.02	15.96 ± 0.33	87.0 ± 0.5	84.5 ± 0.5
F12	1.204 ± 0.114	6.82 ± 1.87	70.21 ± 23.76	89.5 ± 0.5	83.5 ± 0.5
F13	0.786 ± 0.169	19.07 ± 1.44	14.40 ± 12.59	79.0 ± 0.5	70.0 ± 0.5
F14	0.767 ± 0.115	13.46 ± 1.61	12.46 ± 1.14	80.0 ± 0.5	72.0 ± 0.5
F15	0.801 ± 0.364	4.81 ± 2.34	35.74 ± 27.19	72.5 ± 0.5	75.0 ± 0.5
F16	0.947 ± 0.290	5.26 ± 2.90	99.27 ± 26.46	75.0 ± 0.5	81.5 ± 0.5

**Table 4:** Performance of the selected models for the five responses.

Response	Model	Parameters	*R* ^2^	Adjusted *R*^2^	LOO-Predicted *R*^2^	RMSE	AICc
Maximum stress (MPa)	M2	8	0.801	0.627	0.288	0.182 MPa	−28.97
Provisional module (MPa)	M2	8	0.815	0.653	0.341	3.222 MPa	62.92
Elongation (%)	M2	8	0.883	0.780	0.532	12.331%	105.87
Apparent soil mass loos (%)	M1	4	0.673	0.591	0.392	4.465%	54.92
Apparent outdoor mass loss (%)	M2	8	0.881	0.778	0.553	4.071%	70.41

**Table 5:** Predicted individual optima and best observed runs.

Maximized Response	Starch	Gelatin	Temperature (°C)	Time (min)	Predicted Maximum	Best Observed Run
Maximum stress	0.250	0.750	40.0	5.0	1.508 MPa	F04: 1.614 MPa
Provisional modulus	0.250	0.750	40.0	5.0	20.866 MPa	F04: 24.358 MPa
Elongation	0.250	0.750	60.0	10.0	91.626%	F16: 99.269%
Apparent soil mass loss	1.000	0.000	40.0	10.0	89.583%	F09: 93.0%
Apparent outdoor mass loss	1.000	0.000	40.0	5.0	90.350%	F09: 95.0% a 10 min

## Data Availability

The original contributions presented in this study are included in the article. Further inquiries can be directed to the corresponding author.

## Abbreviations

The following abbreviations are used in this manuscript:

DOE	Design of Experiments
ANOVA	Analysis of Variance
ASTM	ASTM International (formerly American Society for Testing and Materials)
PVOH	Poly (vinyl alcohol)
MPa	Megapascal
